# Metabolically Supported Chemotherapy for Managing End-Stage Breast Cancer: A Complete and Durable Response

**DOI:** 10.7759/cureus.14686

**Published:** 2021-04-26

**Authors:** Mehmet Salih İyikesici, Abdul Kadir Slocum, Nasha Winters, Miriam Kalamian, Thomas N Seyfried

**Affiliations:** 1 Medical Oncology, Altınbaş University Bahçelievler Medical Park Hospital, Istanbul, TUR; 2 Medical Oncology, ChemoThermia Oncology Center, Istanbul, TUR; 3 Naturopathic Oncology, Dr. Nasha, Inc., Durango, USA; 4 Nutrition, Dietary Therapies LLC, Hamilton, USA; 5 Biology, Boston College, Boston, USA

**Keywords:** end-stage breast cancer, metabolically supported chemotherapy, ketogenic diet, hyperthermia, hyperbaric oxygen therapy, cancer, warburg effect, repurposed drugs, cancer as a metabolic disease, ketogenic metabolic therapy

## Abstract

Breast cancer accounts for significant morbidity and mortality worldwide. Currently, treatment options in metastatic breast cancer consist of chemotherapy, along with endocrine, radiation, and/or biological therapies. Although advances in management have improved overall survival times, the treatment options for women with end-stage disease are mostly limited to supportive care.

Herein, we present a case report that highlights the response of a 47-year-old premenopausal woman with end-stage (T4N3M1) breast cancer treated with metabolically supported chemotherapy (MSCT), ketogenic diet (KD), hyperthermia (HT), and hyperbaric oxygen therapy (HBOT). The patient first noticed a right breast mass in late 2016, which was initially evaluated and ruled out as a cyst. Skin ulceration was observed in the region of the suspected cyst in May 2017. Subsequent bilateral breast ultrasound identified masses in both breasts and an enlarged right axillary lymph node. The diagnosis following biopsy was grade 3, estrogen receptor-positive (ER+), progesterone receptor-positive (PR+), human epidermal growth factor receptor 2 negative (HER2-), invasive ductal carcinoma of the breast. Initially, the patient refused to receive conventional chemotherapy because of its potential for side effects and toxicity, but she sought medical treatment in August 2018 following extensive disease progression and deterioration of general health. On reevaluation, the patient was considered ineligible for conventional treatment due to her advanced end-stage disease, poor performance status (Eastern Cooperative Oncology Group score: 3), and advanced respiratory symptoms. Exploring other options, the patient was admitted to the ChemoThermia Oncology Center, Istanbul, Turkey in November 2018. At that time, the patient weighed 38 kg (body mass index: 18.1 kg/m^2^) and had extensive metastatic disease with lesions in the brain, lungs, mediastinum, liver, abdomen, and bones that were detected by magnetic resonance imaging of the brain (with contrast) and whole-body (18F)-fluorodeoxyglucose-positron emission tomography-computed tomography. The patient received a six-month treatment protocol comprised of MSCT, KD, HT, and HBOT, which eliminated all detectable lesions. The therapeutic response was sustained for two years with maintenance treatment comprising KD, dietary supplements, and repurposed medications. This single case report presents evidence of a complete and durable response to a treatment protocol combining MSCT and a novel metabolic therapy in a patient with end-stage breast cancer.

## Introduction

Breast cancer is the second most common cancer worldwide, with nearly 2.1 million new patients diagnosed in 2018 globally [1]. It is the fourth leading cause of cancer-related deaths worldwide (627,000 deaths) and the leading cause of cancer-related mortality in women [1]. Breast cancer is a heterogeneous disease with several biologically distinct subtypes. In general, it includes the following three biological subgroups: (1) immunohistochemical expression of the estrogen receptor (ER), (2) amplification of the human epidermal growth factor receptor 2 (HER2) (with or without ER expression), and (3) cancers that do not express ER and HER2 or the progesterone receptor (PR). Despite improvements in technology for early detection, approximately 5% - 7% of women diagnosed with breast cancer have metastatic disease at the time of the first presentation. Additionally, approximately 30% of women initially diagnosed with early-stage, nonmetastatic breast cancer later develop distant metastatic disease despite treatment.

The treatment strategy for breast cancer differs according to the histological type, tumor grade, tumor biology, stage of disease, and clinical factors. Typically, treatment includes a combination of surgery, radiation therapy, endocrine therapy, and chemotherapy. Most women with early-stage breast cancer generally receive local therapies that involve surgery with or without subsequent radiotherapy. Adjuvant systemic therapy is often prescribed to minimize the risk of recurrence. Currently, the mainstay of treatment for patients with metastatic breast cancer involves systemic therapies comprising of chemotherapy, endocrine therapy, and/or biological therapies together with supportive medical care.

In the past few decades, there have been some advancements in the systemic treatment of breast cancer, including new cytotoxic therapies, endocrine therapies, and therapies targeting HER2. Despite these additional treatment options, the median overall survival (OS) for patients with metastatic breast cancer remains low at approximately three years. Unfortunately, survival may not exceed a few months in some cases of aggressive disease [2].

Warburg originally found that aerobic fermentation is a common feature of most cancers [3]. His observation has been confirmed in most malignant cancers and is now referred to as the “Warburg effect.” Metabolic dysregulation in cancer cells is most often characterized by glucose dependency and increased lactate production. Both alterations have been associated with mitochondrial dysfunction [4-5]. This abnormal energy metabolism is evident in fluorodeoxyglucose (FDG)-positron emission tomography (PET) scans, the preferred imaging technique essential to the diagnosis and follow-up protocols used in most systemic cancers, including breast cancer.

Recently, several studies have focused on targeting metabolic aberrations specific to cancer cells, leading to the development of novel chemotherapy applications, namely, metabolically supported chemotherapy (MSCT) [6-12]. In practice, MSCT follows a 14-hour fast starting in the previous evening and administration of pharmaceutical doses of regular insulin prior to the administration of chemotherapy. This strategy aims to increase the efficacy of chemotherapeutic drugs through the following two mechanisms: inducing mild hypoglycemia, leading to acute metabolic stress in cancer cells, and increasing membrane permeability. This is a novel application of chemotherapy that exploits metabolic weakness in cancer cells directly before the application of chemotherapeutic drugs. Because mutations are known to reduce metabolic flexibility in cancer cells, this added stressor can enhance the therapeutic effect of chemotherapy [13]. The glucose dependency of cancer cells also forms the rationale for adopting a diet that decreases circulating glucose levels. A high-fat, carbohydrate-restricted ketogenic diet (KD) reduces blood glucose levels while simultaneously increasing blood ketone levels. Importantly, ketone bodies serve as an alternative energy metabolite to glucose in normal cells but not in cancer cells due to abnormalities in the number, structure, and function of cancer cell mitochondria [14]. Proper administration of a KD can be therapeutic for any cancer that has upregulated the use of glucose for energy [8-13, 15].

Other metabolic treatment modalities also show great potential when used concurrently with KD and MSCT. Hyperthermia (HT) has a direct cytotoxic effect, exploiting the heat sensitivity of cancer cells by increasing the treated tissue temperature to 42°C or higher. HT may also increase the efficacy of chemotherapy and radiotherapy by sensitizing cancer cells to these therapies [7-12, 16]. Hyperbaric oxygen therapy (HBOT) may target cancer cells by increasing oxidative stress. Tumor hypoxia increases the glycolytic dependence of cancer cells while promoting resistance to chemotherapy and radiotherapy. The unfavorable consequences of hypoxia may be counteracted by the administration of oxygen at elevated pressures, resulting in better tissue oxygenation [17]. HBOT increases oxidative stress specifically in tumor cells; when used in combination with chemotherapy and radiotherapy, it enhances therapeutic efficacy in various malignancies [8-12, 16, 18].

Based on this supporting evidence, we hypothesized that MSCT, KD, HT, and HBOT could be effectively combined to target several overlapping metabolic pathways and vulnerabilities of cancer cells. Herein, we describe a case of end-stage metastatic breast cancer where the patient was considered ineligible for the continued standard of care treatment. We show that this patient achieved a complete and durable response to MSCT.

## Case presentation

A 45-year-old premenopausal woman with no identified comorbidities noticed a right breast mass in late 2016, which was subsequently evaluated and ruled out as a cyst by a medical doctor during consultation. At that time, she weighed 54 kg with a body mass index (BMI) of 22.5 kg/m^2^.

**Figure 1:**
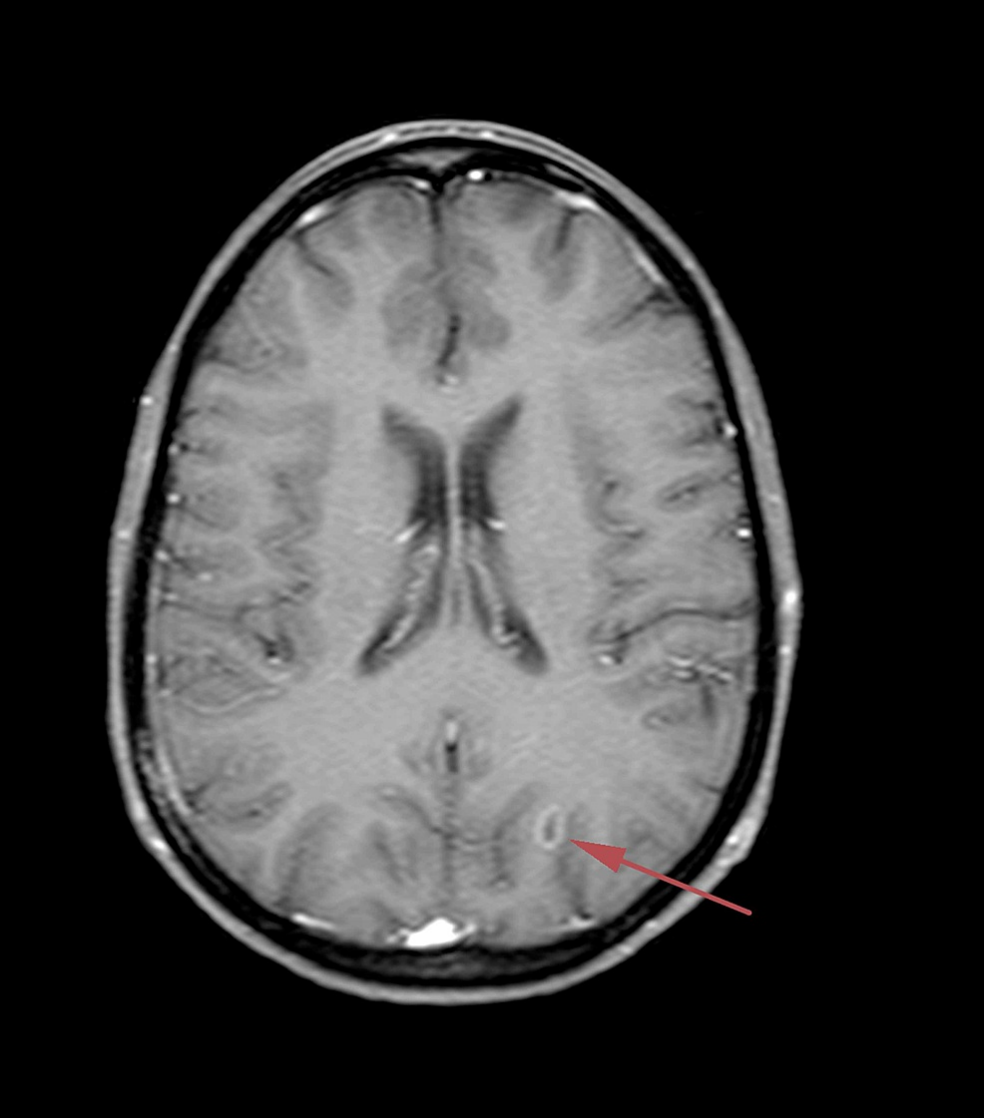
T1-weighted axial brain magnetic resonance image with contrast dated November 5, 2018

**Figure 2:**
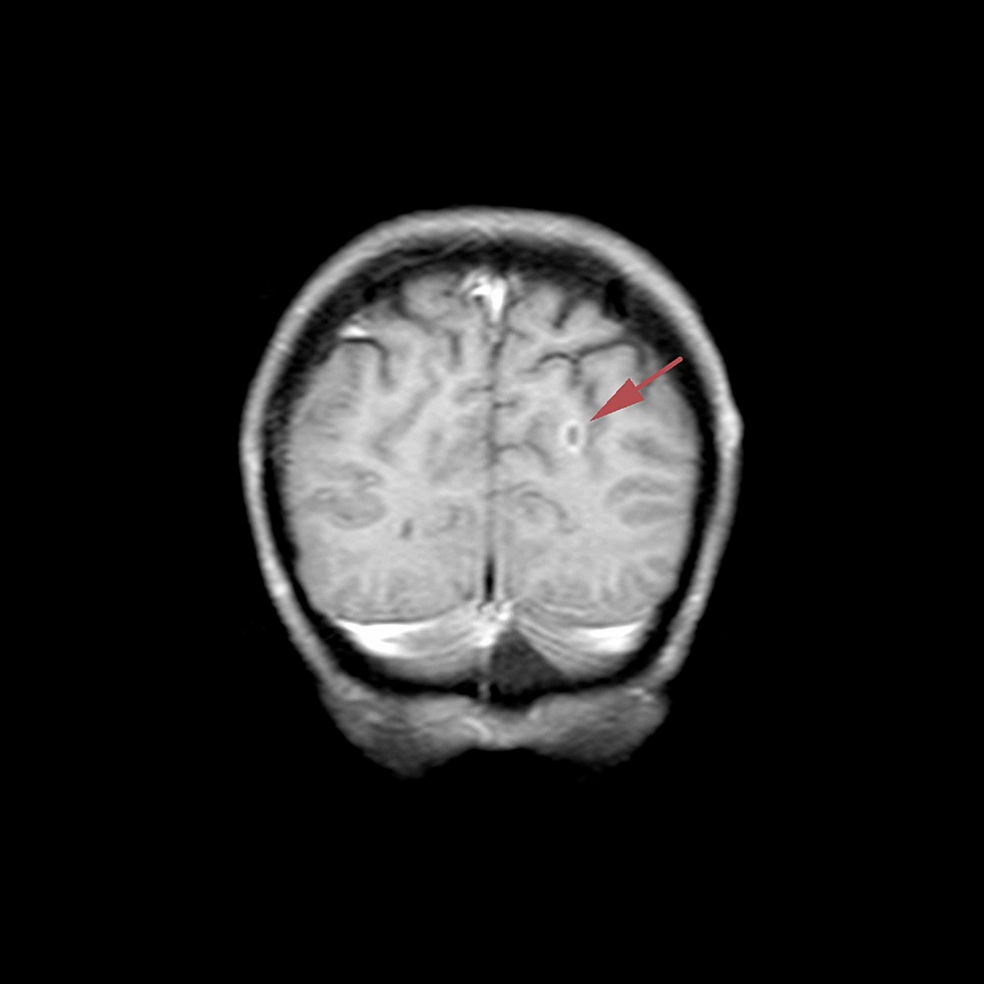
T1-weighted coronal brain magnetic resonance image with contrast dated November 5, 2018

**Figure 3:**
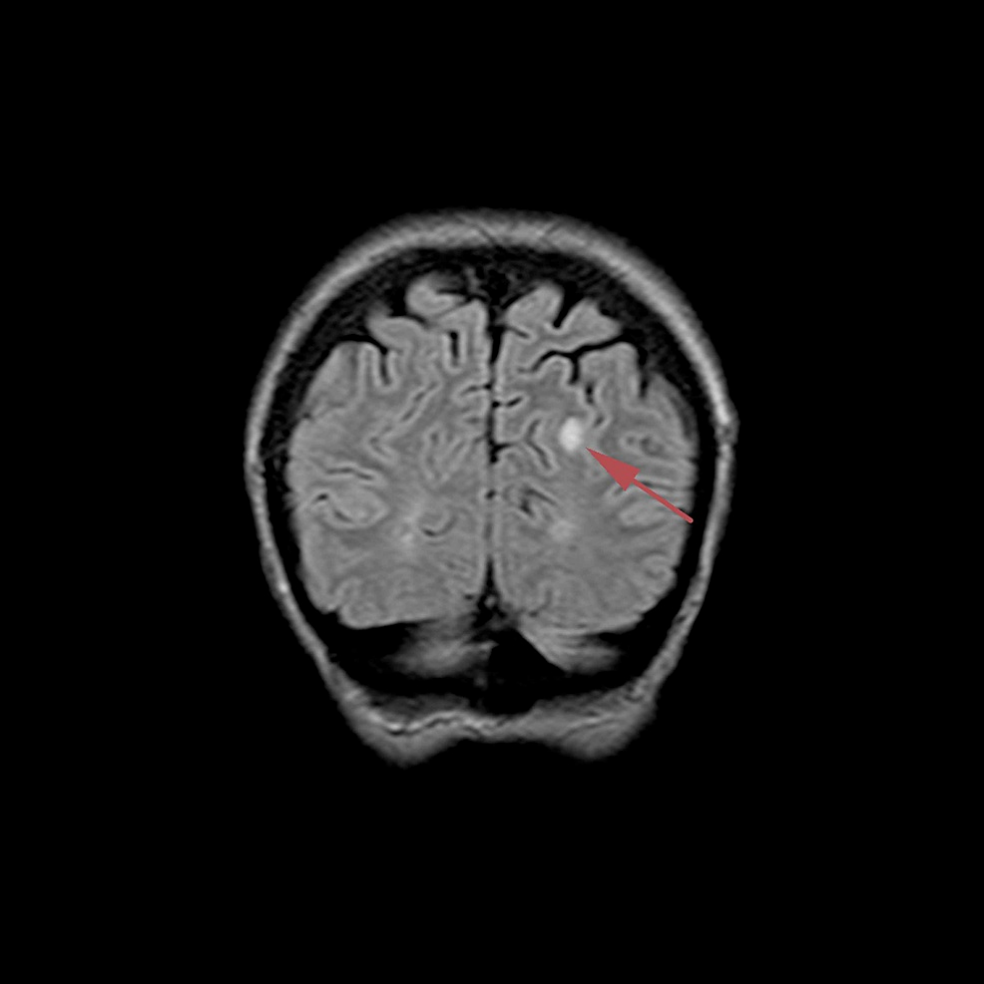
Coronal FLAIR brain magnetic resonance image dated November 5, 2018 where a roughly 8 mm-sized lesion with peripheral contrast enhancement in correlation with a metastatic lesion is seen at the left occipital horn posterior area FLAIR: fluid-attenuated inversion recovery

In May 2017, she was treated for a skin ulceration in the cyst region of her right breast at a hospital near her home in Ohio, USA. The cyst was drained and found to be infected with *Staphylococcus aureus*. She also displayed a palpable nodule near the skin lesion. A bilateral mammogram on May 4, 2017 identified a 1.6-cm mass in the right medial breast with skin tethering. Bilateral breast ultrasound identified masses in both breasts and an enlarged right axillary lymph node. A punch biopsy of the skin lesion was performed on May 11, 2017, which demonstrated a grade 3 invasive ductal carcinoma involving the dermis, epidermis, and dermal lymphatic spaces. ER and PR were strongly positive (> 95%), and HER2 was negative on immunohistochemistry (1+). The patient received antibiotics for her infection and was offered conventional chemotherapy, which she refused because of the chemotherapy’s potential for side effects and toxicity. In March 2018, following clinical and radiological confirmation of disease progression, she received radiotherapy at the University of California, Los Angeles for local disease control. Subsequent evaluation in June 2018 using whole-body (18F)-FDG-PET-computed tomography (PET-CT) scan identified widespread metastatic disease. FDG-avid lymphadenopathy (LAP) was detected in the lower-left neck and supraclavicular region. Locally advanced breast cancer was suggested based on the findings of a large ulcerative lesion with intense hypermetabolism in irregular soft tissue thickening involving the medial aspect of both breasts. Involvement of the right adjacent pectoral musculature and direct invasion of the sternum were the areas of concern. The possibility of superimposed inflammatory/infectious processes (including osteomyelitis of the sternum) and postradiation changes were also considered. Numerous other FDG-avid lesions in both breasts and soft tissues of the chest wall were suspected to be metastases and/or multifocal primary lesions. Multiple FDG-avid pulmonary (and possibly pleural) metastases and extensive metastatic LAP were also detected. Multiple FDG-avid metastatic liver lesions and portacaval adenopathy were identified in the abdomen and pelvis, along with multifocal osseous metastases in the axial and appendicular skeleton. In July 2018, a new ultrasound-guided core biopsy was performed on the left breast axillary, which further documented widespread metastatic disease. Evidence of grade 2 invasive mammary carcinoma (ER (+), PR (+), and HER2 (-)) with mixed ductal and lobular features was consistent with her initial biopsy from May 2017. A follow-up CT scan in August 2018 confirmed significant and wide interval progression of metastatic disease at multiple pre-identified sites. An interval increase in the size of the pericardial effusion caused narrowing of both atria, particularly the left atria. Following this dismal report, the patient underwent echocardiography to assess tamponade physiology, which was reported to correlate with the PET-CT scan findings. Pericardiocentesis was performed with subsequent drainage of 360-mL of serosanguinous fluid. The patient was subsequently considered ineligible for further conventional therapy due to her advanced end-stage disease, poor performance status (Eastern Cooperative Oncology Group [ECOG] score: 3), and advanced respiratory symptoms. She obtained a second opinion and was informed that her expected prognosis was less than one month. The patient sought other treatment options and was ultimately admitted to the ChemoThermia Oncology Center, Istanbul, Turkey on November 5, 2018. Upon admission, her weight was 38 kg (BMI: 18.1 kg/m^2^). Poor performance status (ECOG score: 3) and a recent worsening respiratory tract infection were also noted. She underwent an objective clinical evaluation and documentation of her status, which included magnetic resonance imaging (MRI) of the brain with contrast, whole-body FDG-PET CT, and a detailed laboratory assessment (Figures 1-3). The MRI revealed an 8-mm malignant lesion in the left occipital lobe. The FDG-PET/CT scan reconfirmed widespread metastatic disease with progression when compared to her prior scan. Laboratory results showed normal kidney function, a slight elevation in the liver enzyme level, and high C-reactive protein level of 119 mg/mL. Tumor markers were elevated with carcinoembryonic antigen and cancer antigen 15-3 of 45.6 ng/mL and 527 U/mL, respectively.

**Figure 4:**
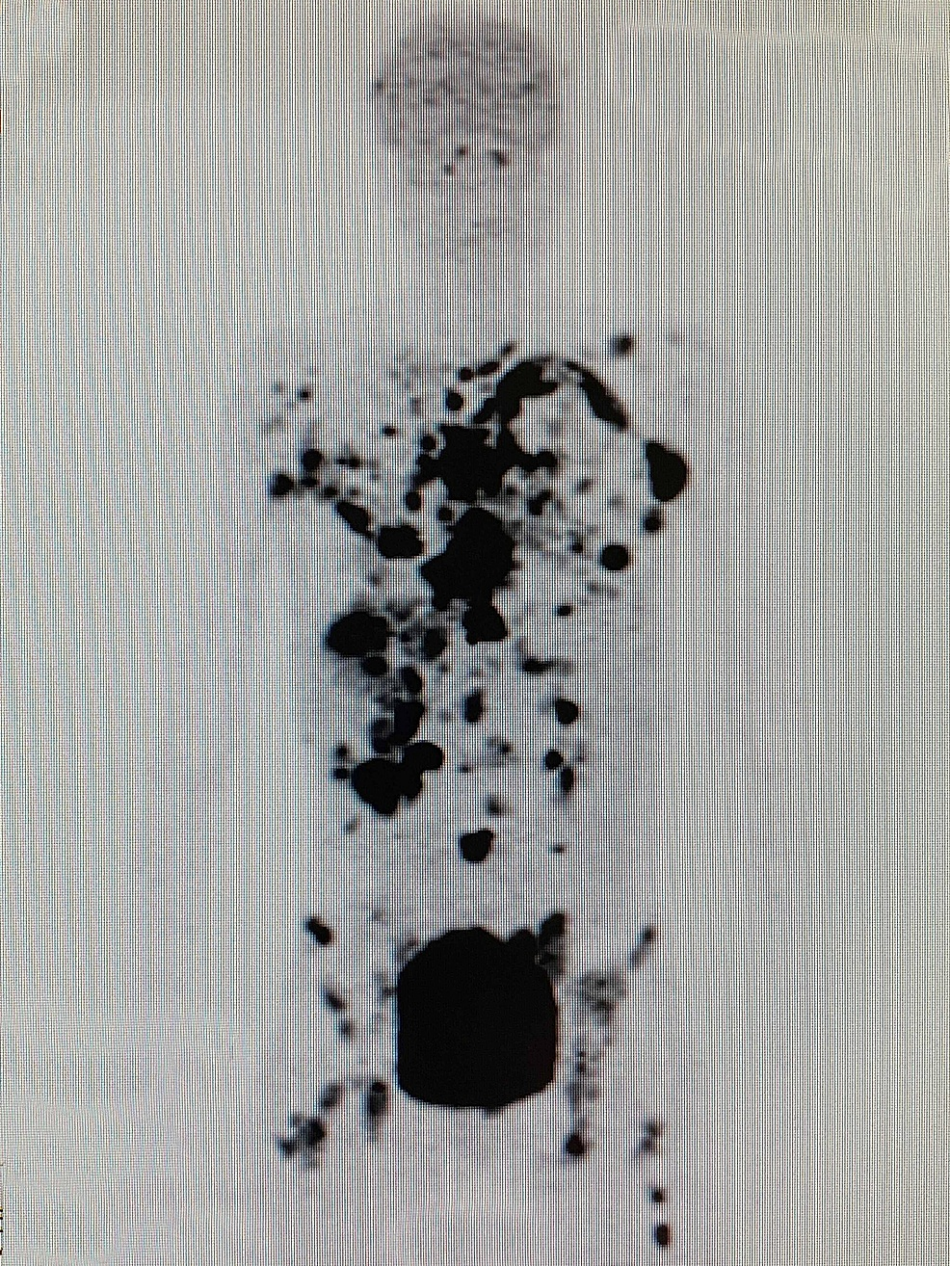
Whole body (18F)-FDG-PET/CT scan done before treatment on November 5, 2018 showing widespread multiple metastatic lesions at the lungs, mediastinum, liver, abdomen and bones. (18F)-FDG-PET/CT: 18F-fluorodeoxyglucose positron emission tomography/computed tomography

Whole-body (18F)-FDG-PET-CT scan performed before treatment on November 5, 2018 showed widespread multiple metastatic lesions in the lungs, mediastinum, liver, abdomen, and bones (Figure 4, Video 1).

**Video 1:** Coronal fusion video of the whole body (18F)-FDG-PET/CT scan done before treatment on November 5, 2018 (18F)-FDG-PET/CT: 18F-fluorodeoxyglucose positron emission tomography/computed tomography

Following this detailed physical, biochemical, and radiological evaluation confirming widespread end-stage disease with a poor prognosis (< 1 month), the patient received counseling regarding potential side effects and complications of treatment. After providing written informed consent, the patient started treatment at our clinic on November 7, 2018.

Metabolically supported chemotherapy

Premedication consisted of 45.5 mg of pheniramine maleate, 0.25 mg of palonosetron hydrochloride, and regular insulin (Humulin® R) in doses ranging between 5 and 10 IU to attain mild hypoglycemia, defined as blood glucose levels of approximately 50 - 60 mg/dL (in accordance with MSCT protocols) [6-12]​​​​. The patient received 17 treatment sessions in each session after 14-hour fasting. The patient’s blood glucose levels were measured by finger stick testing upon admission, and her blood glucose was titrated to the targeted pretreatment mildly hypoglycemic level with insulin administration. The attending physician and nurse closely monitored the patient’s blood glucose levels and assessed her for clinical symptoms of hypoglycemia. Her fasting blood glucose levels upon admission ranged between 67 and 88 mg/dL, whereas her achieved pretreatment blood glucose ranged between 51 and 58 mg/dL. A chemotherapy regimen comprised of gemcitabine (600 mg/m^2^), carboplatin area under the curve 2, and paclitaxel (60 mg/m^2^) was initiated upon reaching the targeted blood glucose level. This drug combination was delivered on the first and eighth day of a 21-day cycle. Concurrent with this treatment, 4 mg of zoledronic acid was delivered every 28 days to treat the metastatic bone lesions.

Ketogenic diet, hyperthermia, and hyperbaric oxygen therapy

The patient was advised to adopt a diet that was high in fat and significantly limited in carbohydrates, modeling a KD. She and her caregiver were informed in detail about which foods to include and which foods should be strictly avoided. She was directed to modify familiar meals by eliminating grains and reducing sugars and dense starches while simultaneously incorporating more fats. Given that her cancer was ER +, she was advised to limit high-fat dairy products, which are contraindicated in estrogen-sensitive cancers. At each visit, an assessment of the patient’s blood glucose levels and review of the caregiver’s food records offered evidence of the patient’s ongoing compliance with KD principles, and further refinements to the dietary plan were made at each visit. Together with every MSCT treatment, the patient also received local HT and HBOT: two sessions of each - one of each on the same day as MSCT and one of each on the day following MSCT.

For each 60-minute HT session, an OncoTherm EHY-3010 HT device (OncoTherm, Troisdorf, Germany) was used to gradually increase the temperature of the tumoral region. The OncoTherm device uses modulated electrohyperthermia technology to selectively increase metabolic pressure on tumor tissue without negatively affecting the normal surrounding tissues, including the skin. A 30 × 40-cm mobile electrode was used for HT sessions and was placed starting from the supraclavicular region and covering the thoracic region and the upper abdomen, including the liver, targeting both the primary tumor and the supraclavicular, thoracic, and liver metastatic lesions. The objective of HT was twofold: (1) to obtain a tumoral tissue temperature greater than 43°C at the tumor site, as this temperature is reported to be cytotoxic, and (2) to increase blood circulation to the tumoral region which, in turn, is expected to improve chemotherapy drug delivery. The device used indirect temperature estimations based on the energy applied: indirect heat estimation for this patient’s local HT sessions ranged from 44.1°C to 45.6°C.

For each 60-minute HBOT session, a Quamvis^320®^ hyperbaric oxygen chamber (OxyHealth, Santa Fe Springs, CA, USA) was used. This is a soft-walled chamber with a 32-inch diameter when inflated. The chamber was pressurized using a clean air compressor that filters air to 0.01 µm and a 10 L/min O_2_ concentrator to produce an operating pressure of 1.5 atmospheres absolute.

**Figure 5:**
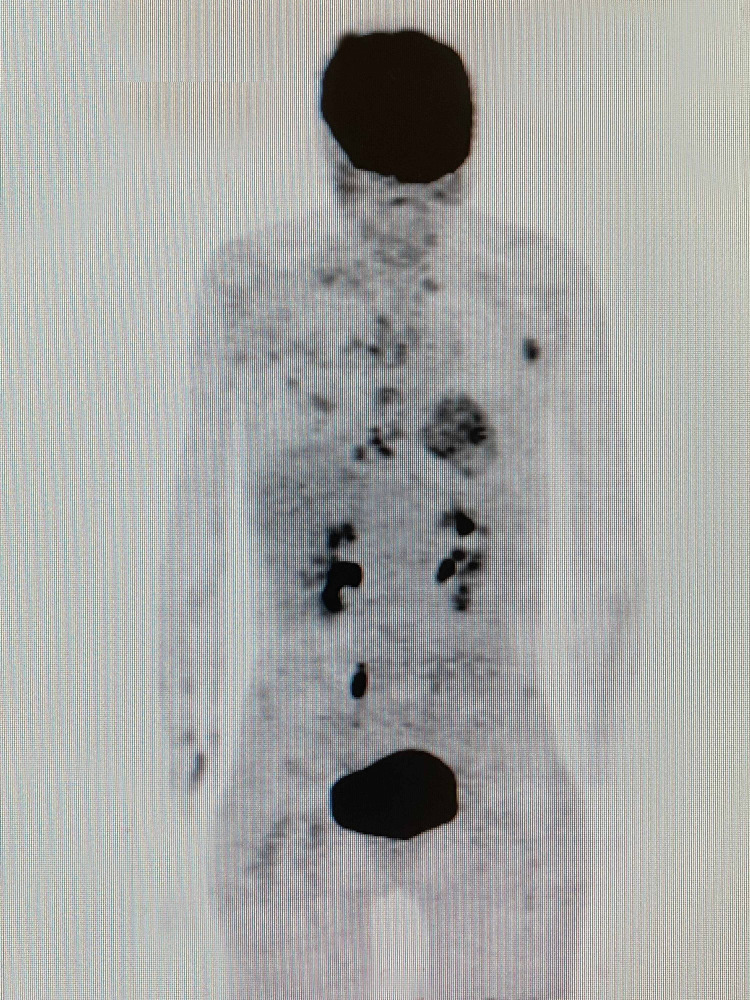
Whole body (18F)-FDG-PET-CT scan done after four cycles of MSCT, KD, HT, and HBOT treatment (January 16, 2019) showing a partial response to treatment of the metastatic liver lesions and left axillary LAP and complete response to treatment at all other lesions (18F)-FDG-PET/CT: 18F-fluorodeoxyglucose positron emission tomography/computed tomography; HBOT: hyperbaric oxygen therapy; HT: hyperthermia; KD: ketogenic diet; MSCT: metabolically supported chemotherapy

The patient’s treatment protocol comprising of MSCT, KD, HT, and HBOT was started together with infusional antibiotics on November 7, 2018. On November 9, she was hospitalized with respiratory complaints. On November 10, following the worsening of her respiratory function as evidenced by low blood oxygen saturation levels of 50% - 60%, she was intubated for effective medical support and transferred to the intensive care unit. Upon admission to the intensive care unit, her rectal swab culture was positive for vancomycin-resistant *Enterococcus*. Following the routine hospital protocol, she was moved to isolation within the intensive care unit, and her treatment was adjusted accordingly. She received mechanical ventilatory support for five days, along with infusional supportive care and antibiotic therapy. She was extubated on November 15 and transferred from the intensive care unit to the internal medicine ward where her infusional supportive care and antibiotic therapy were continued for an additional five days. The patient was discharged from inpatient hospital care on November 19 with stable vital signs and an oxygen saturation of 98% at room air. Her treatment was resumed as an outpatient at our clinic on the same day. After completing her fourth treatment cycle, a treatment response evaluation PET-CT scan was performed on January 16, 2019. Her PET-CT scan revealed the following findings: no pathologic FDG uptake at the head and neck; FDG uptake at both supraclavicular areas within physiological limits; 14-mm hypermetabolic LAP (standardized uptake value (SUV): 4 - 5) at the left axilla, and no mass lesion with FDG uptake in the left and right breast parenchymal areas. The thorax scan, when compared to her prior scan, revealed fewer and smaller nodules without FDG uptake. Free pleural fluid covering 3 cm on the right and 2.5 cm on the left was observed. In the mediastinal and hilar regions, no lymph nodes with pathologic FDG uptake were evident. The previously identified nodules in the liver parenchyma were again evident in the abdominal scan, with a significant reduction in metabolic activity and size, with the largest nodule measuring 3.2 cm at segment 8 (SUV: 3-4). Sclerotic changes without FDG uptake were observed in the skeletal system scan. In summary, the scan documented a treatment response at all sites. Low-density FDG uptake at nodules in the liver and a left axillary LAP indicated a partial response at these sites with a complete response to treatment at all other lesions (Figure 5, Video 2).

**Video 2:** Coronal fusion video of the whole body (18F)-FDG-PET-CT scan done following four cycles of MSCT, KD, HT, and HBOT treatment on January 16, 2019 (18F)-FDG-PET/CT: 18F-fluorodeoxyglucose positron emission tomography/computed tomography; HBOT: hyperbaric oxygen therapy; HT: hyperthermia; KD: ketogenic diet; MSCT: metabolically supported chemotherapy

**Figure 6:**
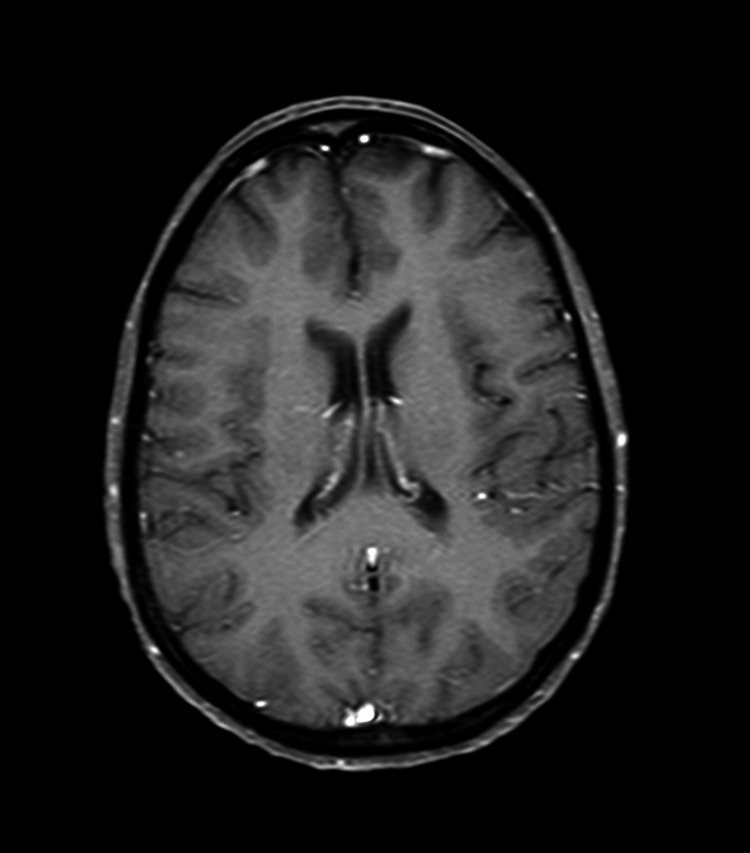
T1-weighted axial brain magnetic resonance image with contrast on April 25, 2019

**Figure 7:**
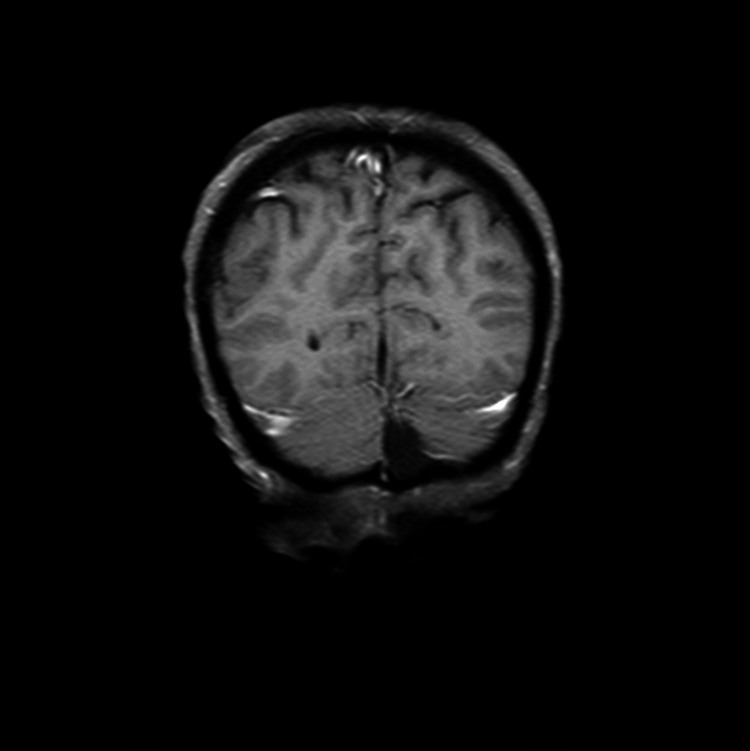
T1-weighted coronal brain magnetic resonance image with contrast on April 25, 2019

**Figure 8:**
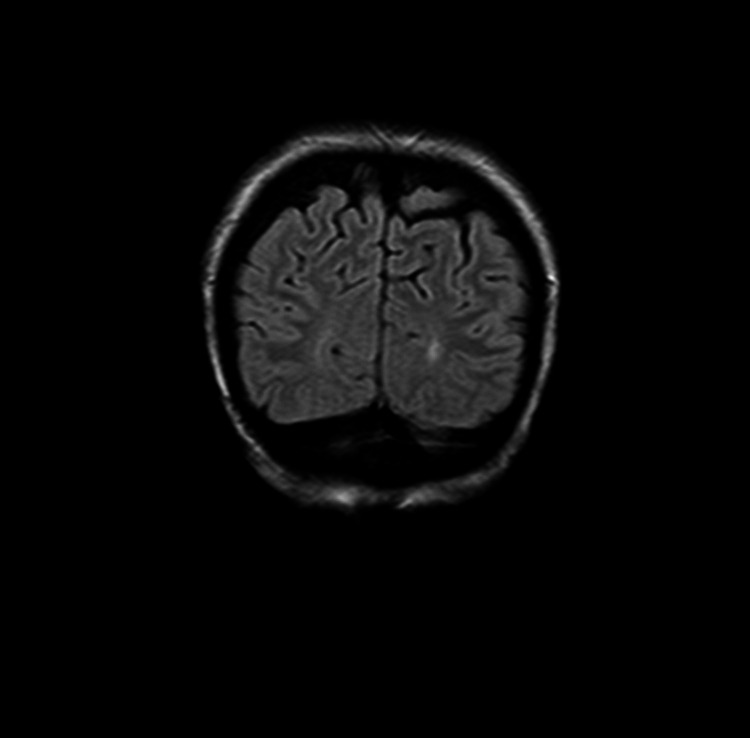
Coronal FLAIR brain magnetic resonance image on April 25, 2019 with the same sequences as that of the brain MRI performed on November 5, 2018 reveals a roughly 8-mm-sized lesion with peripheral contrast enhancement in correlation with a metastatic lesion that was no longer at the left occipital horn posterior area FLAIR: fluid-attenuated inversion recovery

**Figure 9:**
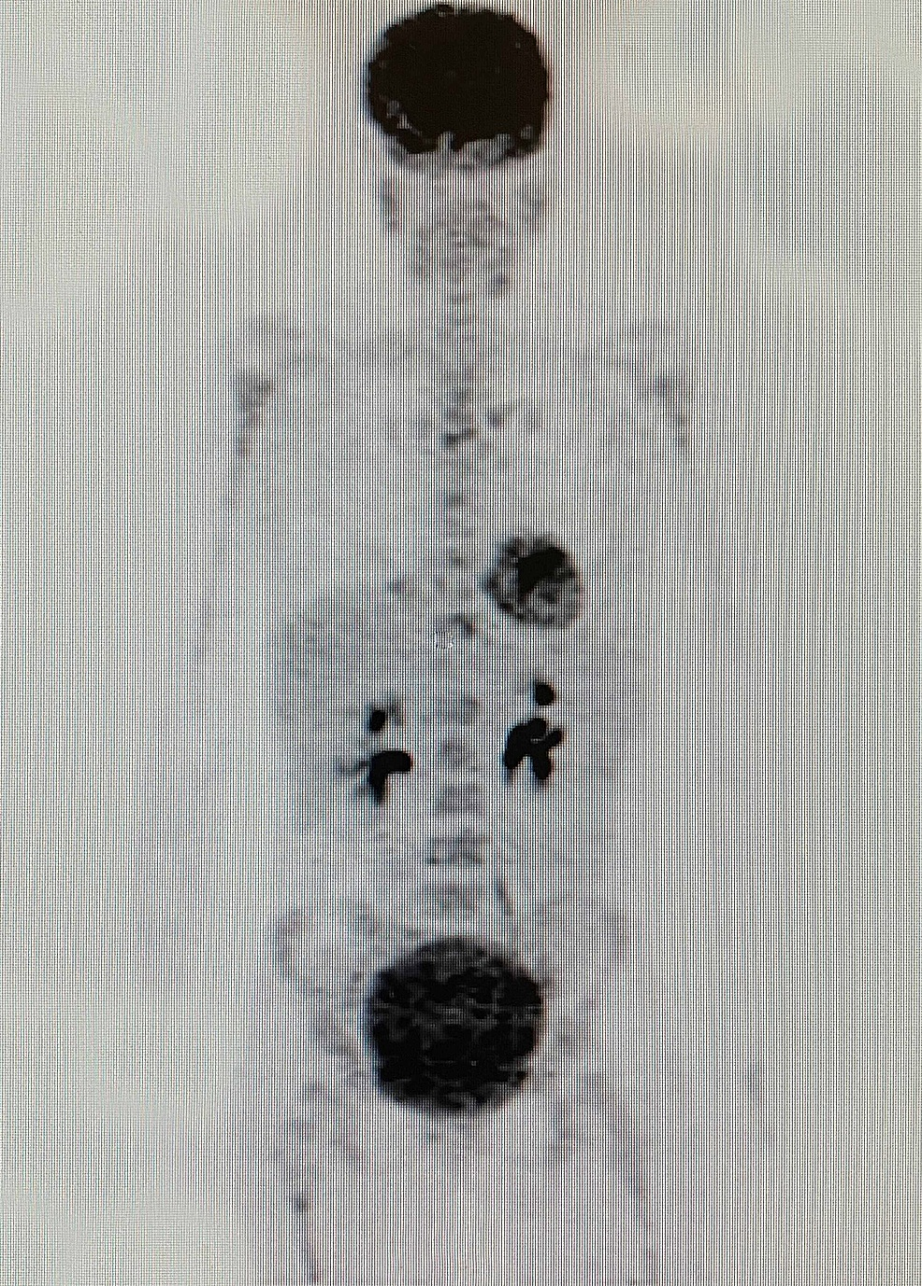
Whole body (18F)-FDG-PET-CT scan done on April 25, 2019 after nine cycles of MSCT, KD, HT, and HBOT treatment showing no pathological FDG uptake, indicative of a complete response (18F)-FDG-PET/CT: 18F-fluorodeoxyglucose positron emission tomography/computed tomography; HBOT: hyperbaric oxygen therapy; HT: hyperthermia; KD: ketogenic diet; MSCT: metabolically supported chemotherapy

Following the PET-CT scan on January 16, 2019, the patient continued with the same treatment protocol for an additional five cycles of treatment. A follow-up PET-CT scan and contrast MRI of the brain were performed on April 25, 2019, for treatment response evaluation. When compared to the January scan, the April scan revealed that the multiple nodules in the liver no longer displayed malignant FDG uptake. Similarly, the hypermetabolic activity previously observed in the left axillary LAP was no longer evident. The April MRI scan of the brain showed a complete response to treatment with no evidence of an 8-mm malignant lesion previously documented in the pretreatment MRI dated November 2018. In summary, the scan reports from April indicated a complete response to treatment for all lesions with no evidence of active disease (Figures 6-9, Video 3).

**Video 3:** Coronal fusion video of the whole body (18F)-FDG-PET-CT scan done on April 25, 2019 following nine cycles of MSCT, KD, HT, and HBOT treatment (18F)-FDG-PET/CT: 18F-fluorodeoxyglucose positron emission tomography/computed tomography; HBOT: hyperbaric oxygen therapy; HT: hyperthermia; KD: ketogenic diet; MSCT: metabolically supported chemotherapy

After treatment and before returning home to Ohio, the patient was advised to continue the KD, along with previously prescribed medications (metformin, doxycycline, meloxicam, capecitabine, rivaroxaban) and dietary supplements (medicinal mushrooms, curcumin, quercetin, alpha-lipoic acid, selenium, milk thistle, resveratrol) as follow-up maintenance therapy. To date (two years), she has continued with the recommended maintenance protocol in Ohio. Routine follow-up scans (PET/CT and MRI) and laboratory tests were performed and assessed by a local medical oncologist with some gaps due to limitations imposed by COVID-19. Her most recent follow-up PET/CT scan and laboratory tests were performed in March 2021 and indicated stability with no evidence of recurrent disease. The patient’s quality of life improved, and she was able to resume meaningful full-time work beginning May 1, 2019.

## Discussion

Herein, we report the case of a 47-year-old woman from Ohio, USA with stage IV (T4N3M1) grade 3, ER +, PR +, and HER2- breast cancer that had metastasized to the brain, lungs, mediastinum, liver, abdomen, and bones, who was ineligible for standard conventional treatment due to advanced disease, poor performance status, and life expectancy of less than one month. She was admitted to the ChemoThermia Oncology Center, Istanbul, Turkey, where she received MSCT, KD, HT, and HBOT, along with supportive medications and dietary supplements, resulting in a complete and enduring response to treatment spanning two years at present. Our findings support the potential benefit of integrating a combination of modalities targeting multiple vulnerabilities of tumor cells with standard chemotherapeutic drugs administered using our non-standard MSCT protocol. We hypothesized that the efficacy and tolerability of standard chemotherapeutic drugs would be enhanced when delivered while fasting and with insulin-potentiated therapy, combined with other low-toxicity therapies that target the metabolic vulnerabilities of cancer cells. With disease regression as the goal, this patient was able to achieve a complete and durable response to the administered treatment. In addition, the patient’s quality of life was enhanced following each treatment cycle, and her performance status was ECOG 0-1 at the completion of her treatment in Istanbul.

Previous studies with MSCT regimens that have also reported encouraging findings in patients with various types of malignancies include five retrospective case series [6, 9-12]​​​​​​ and two case reports [7-8]. A recent study evaluated the efficacy and tolerability of weekly carboplatin/paclitaxel combination treatment administered in a metabolically supported manner in patients with metastatic non-small cell lung carcinoma (NSCLC), reporting mean OS of 42.9 months compared to survival times with standard conventional treatment ranging from 6.3 to 11.3 months [9]. Another recent study comprised of patients with metastatic pancreatic cancer that examined the efficacy of a gemcitabine‑based and/or FOLFIRINOX protocol administered in a metabolically supported protocol reported an encouraging median survival of 15.8 months compared to the poorer prognosis typical for this cancer [10]. Similarly, a study evaluating the outcomes of a taxane-, platinum-, and fluoropyrimidine‑based combination chemotherapy protocol applied in a metabolically supported manner in patients with advanced gastric cancer reported promising outcomes with a mean survival time of 39.5 months compared to survival times ranging from 9.2 to 14.6 months in standard conventional treatment [11]. In an 81-year-old patient with locally advanced rectal cancer, the FOLFOX-6 regimen was administered using the MSCT approach and was proven to be tolerable and effective as it provided complete clinical and pathological responses [7]​​​​​​​. In another case, a 29‑year‑old woman with stage IV (T4N3M1) triple-negative breast cancer achieved a complete clinical, radiological, and pathological response using an MSCT regimen combining docetaxel, doxorubicin, and cyclophosphamide [8]​​​​​​.

In this metabolically supported approach, several mechanisms may act to improve the efficacy of chemotherapy. The 14-hour fast prior to chemotherapy, insulin-induced mild hypoglycemia, and insulin itself may have been central to the observed outcomes. Fasting lowers blood glucose levels and sensitizes cancer cells to treatment by increasing metabolic stress in cancer cells while also possibly protecting normal cells from chemotoxicity, thereby improving tolerance to treatment [19-20]​​​​​​​. In addition, insulin-induced mild hypoglycemia targets glucose dependency and dysregulated energy metabolism in cancer cells, thus further reducing the availability of glucose. This therapeutic strategy may cause acute metabolic stress in cancer cells, thus making them more vulnerable to the cytotoxicity of chemotherapeutic agents [3-4]​​​​​​​. Insulin may also facilitate the action of chemotherapeutic agents at the cellular level by increasing membrane fluidity and permeability. The subsequent formation of drug-insulin complexes internalized by receptor-mediated endocytosis may facilitate an increase in drug penetration, thereby further enhancing the cytotoxic effects of chemotherapeutic agents. A number of insulin and insulin-like growth factor (IGF) receptors are upregulated on tumor cell membranes when compared to healthy cells of similar tissue origin. Breast cancer cells, for example, have approximately seven times more insulin receptors and 10 times more IGF receptors than normal breast tissue. The response of these receptors to insulin potentially extends the S phase of the cell cycle, thereby rendering cancer cells more susceptible to the cytotoxic effects of chemotherapeutic agents for longer periods of time. In addition, the lower concentration of insulin and IGF receptors on normal cells may effectively spare them from the cytotoxic effects of chemotherapeutic agents, possibly resulting in enhanced safety and tolerability.

Another component of our combinatorial treatment protocol was the KD, which also exploits the metabolic dysregulation of tumor cells, possibly exerting its action by lowering the circulating glucose level while increasing the ketone body level. Although the KD has been used for decades as a treatment for intractable pediatric epilepsy, its potential as a therapeutic modality that targets the energy metabolism of cancer cells has only recently been explored. To date, several preclinical studies and case reports have provided support for its potential adjunctive use in cancer treatment [8-12, 15]​​​​​​​. Notably, the patient described in this study did not receive radiotherapy for brain metastasis. The standard application of the chemotherapeutic drugs used in this protocol was not expected to produce a response due to the limited penetration of drugs across the blood-brain barrier. Instead, the resolution of her brain lesion might be attributed to ketogenic metabolic therapy, which has been reported to regress brain lesions in many preclinical glioma models and in patients with glioblastoma multiforme.

HT and HBOT also target dysregulated energy metabolism in cancer cells. HT exploits the heat sensitivity of cancer cells and is cytotoxic at temperatures > 43°C. Besides having a direct cytotoxic effect, HT contributes to therapeutic efficacy by facilitating chemotherapeutic drug uptake, inhibiting deoxyribonucleic acid (DNA) repair in cancer cells, and increasing oxygen radical production [16]​​​​​​​. In this patient, tumor hypoxia, which is known to contribute to tumor aggressiveness and resistance to chemotherapy and radiotherapy, was targeted with HBOT. Both HT and HBOT also target the reliance of tumor cells on glycolysis, which is associated with the upregulation of antioxidant activity responsible for the increased resistance of the tumor to prooxidant chemotherapy and radiation therapies [13]. As a consequence, HT and HBOT selectively increase oxidative stress in tumor cells, thus working synergistically with prooxidant chemotherapy and radiotherapy. Ketone bodies protect normal cells from oxidative stress and provide a substrate for energy production in most normal cells. The concomitant synergistic use of these therapies (fasting, KD, HT, HBOT) and their potential to increase the efficacy of conventional therapies have already been reported in several previous studies on various malignancies [4, 8-12, 16, 18]​​​​​​​. In a recent study by Ohguri et al., patients with NSCLC with multiple pulmonary metastases received a carboplatin/paclitaxel chemotherapy regimen, alongside HT and HBOT; reported results suggested that this combination offered a feasible and potentially efficacious protocol [16]​​​​​​​. In addition, promising outcomes, including remarkably improved survival rates, were reported in four recent case series evaluating the efficacy of the concurrent administration of these three modalities together with MSCT in patients with stage IV lung, stage IV pancreatic, stage III-IV gastric, and stage II-IV rectal cancers [9-12]​​​​​​​.

The patient in this case report had stage IV (T4N3M1) grade 3, ER +, PR +, HER2- breast cancer that had metastasized to the brain, lungs, mediastinum, liver, abdomen, and bones. The therapeutic strategy of combining MSCT, KD, HT, and HBOT administered over a six-month period yielded a complete response that has been sustained for two years with maintenance treatment consisting of KD, dietary supplements, and repurposed medications.

## Conclusions

Clearly, there are many obstacles and failures in the current conventional clinical approach for treating highly metastatic end-stage disease. The findings of this study, along with the growing body of preclinical and clinical evidence, demonstrate that it is feasible to implement more effective and less toxic individualized cancer treatment protocols. To this end, more studies are needed to explore non-drug modalities and combination therapies that target multiple metabolic and cellular vulnerabilities in cancer cells. This case report represents remarkable changes in how we view cancer’s vulnerabilities and a significant leap forward from the current conventional model.
